# EGFR mediates activation of RET in lung adenocarcinoma with neuroendocrine differentiation characterized by ASCL1 expression

**DOI:** 10.18632/oncotarget.15676

**Published:** 2017-02-24

**Authors:** Kaustubh Bhinge, Lin Yang, Simone Terra, Aqsa Nasir, Prasuna Muppa, Marie Christine Aubry, Joanne Yi, Nafiseh Janaki, Irina V. Kovtun, Stephen J. Murphy, Geoffrey Halling, Hamed Rahi, Aaron Mansfield, Mariza de Andrade, Ping Yang, George Vasmatzis, Tobias Peikert, Farhad Kosari

**Affiliations:** ^1^ Department of Molecular Medicine, Mayo Clinic, Rochester, MN, USA; ^2^ Department of Laboratory Medicine and Pathology, Mayo Clinic, Rochester, MN, USA; ^3^ Department of Molecular Pharmacology and Experimental Therapeutics, Mayo Clinic, Rochester, MN, USA; ^4^ Department of Health Sciences Research, Mayo Clinic, Rochester, MN, USA; ^5^ Department of Medical Oncology, Mayo Clinic, Rochester, MN, USA; ^6^ Department of Anatomic Pathology, Mayo Clinic, Rochester, MN, USA; ^7^ Department of Pulmonary and Critical Care Medicine, Mayo Clinic, Rochester, MN, USA

**Keywords:** ASCL1, RET, EGFR, lung cancer, neuroendocrine

## Abstract

Achaete-scute homolog 1 (ASCL1) is a neuroendocrine transcription factor specifically expressed in 10-20% of lung adenocarcinomas (AD) with neuroendocrine (NE) differentiation (NED). ASCL1 functions as an upstream regulator of the RET oncogene in AD with high ASCL1 expression (A^+^AD). RET is a receptor tyrosine kinase with two main human isoforms; RET9 (short) and RET51 (long). We found that elevated expression of RET51 associated mRNA was highly predictive of poor survival in stage-1 A^+^AD (p=0.0057). Functional studies highlighted the role of RET in promoting invasive properties of A^+^AD cells. Further, A^+^AD cells demonstrated close to 10 fold more sensitivity to epidermal growth factor receptor (EGFR) inhibitors, including gefitinib, than AD cells with low ASCL1 expression. Treatment with EGF robustly induced phosphorylation of RET at Tyr-905 in A^+^AD cells with wild type EGFR. This phosphorylation was blocked by gefitinib and by siRNA-EGFR. Immunoprecipitation experiments found EGFR in a complex with RET in the presence of EGF and suggested that RET51 was the predominant RET isoform in the complex. In the microarray datasets of stage-1 and all stages of A^+^AD, high levels of EGFR and RET RNA were significantly associated with poor overall survival (p < 0.01 in both analyses). These results implicate EGFR as a key regulator of RET activation in A^+^AD and suggest that EGFR inhibitors may be therapeutic in patients with A^+^AD tumors even in the absence of an *EGFR* or *RET* mutation.

## INTRODUCTION

Lung cancer is the most common cause of cancer related deaths in men and women in the United States. Non-small cell lung cancers (NSCLCs) account for more than 85% of the total number of lung cancer cases diagnosed every year; of these, the adenocarcinoma (AD) subtype alone accounts for about 40% of cases [1]. In an effort to identify “driver” mutations, high throughput sequencing of lung tumors has been undertaken by major cancer centers in the US and across the globe. These investigations have uncovered important oncogenic mutations, such as *EGFR* in about 10-15% of AD population in the US. However, in close to 45% of cases driver mutations in lung ADs are still unknown.

Previously, we reported that in 10-20% of lung AD the expression of achaete-scute homolog-1 (ASCL1 or Mash1) was elevated [2]. ASCL1 is a neuroendocrine transcription factor belonging to the basic helix-loop-helix (bHLH) family and is indispensable for the development of lung neuroendocrine cells [2]. Importantly, ASCL1 was found to be the regulator of the RET oncogene in AD cells with high ASCL1 expression (A^+^AD) by sh-RNA [2] and ChIP-seq experiments [3]. Furthermore, levels of *RET* mRNA in tumors from A^+^AD patients had significant association with the overall survival (OS) in a large cohort of stage-1 AD microarray dataset from multiple institutions. These findings suggested that targeting RET can provide potential therapeutic benefits in patients with A^+^AD.

In this study, we examined the potential role of wild type RET in influencing the oncogenic properties of A^+^AD tumors. Additional effort was made to identify drugs that could selectively target RET signaling and examined the role of RET isoform separately. Two main transcript variants of RET are expressed in humans, variant 2 (NM_020975.4) corresponding to RET51 known as the long protein isoform and variant 4 (NM_020630.4) corresponding to RET9 known as the short protein isoform. The two isoforms share 100% homology in the first 1063 residues. However, the flanking c-terminal residues are different in RET9 and RET51, having 9 and 51 amino acids, respectively [4]. This study corroborated our previous finding about the influence of RET expression on patient outcomes and also identified significant interaction between RET and EGFR, which was inhibited by EGFR inhibitors. We also found significant associations between levels of *EGFR* and *RET* transcripts and patient overall survival in A^+^AD patients. Our findings may have significant implications regarding the role of EGFR inhibitors in the treatment of A^+^AD patients, even if these tumors do not carry an *EGFR* mutation.

## RESULTS

### Associations of RET mRNA splice variants with the overall survival of stage-1 A^+^AD patients

**Figure 1:**
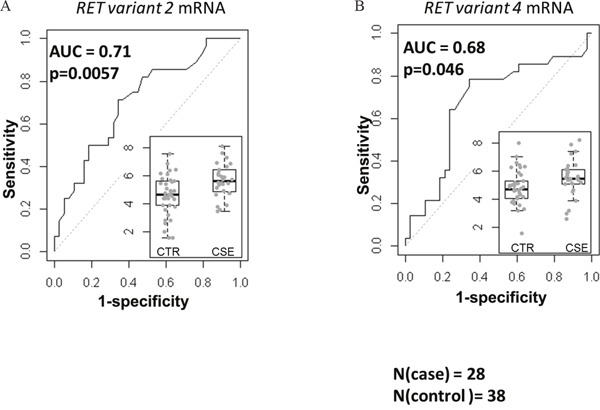
Associations of mRNA corresponding to the two RET isoforms with the OS based on the area under receiver operating characteristics (AUC) The left **(A)** and the right **(B)** panels are AUC for variant 2 (corresponding to the *RET51*) and variant 4 (corresponding to *RET9*). The inlets are normalized expression levels in controls (CTR, n=38) and cases (CSE, n=28). *RET51* splice variant was highly predictive of OS (p=0.0057) and the association of *RET9* with the OS was marginal (p=0.046).

Previously, we reported that the expression of RET mRNA was predictive of overall survival (OS) in stage-1 A^+^AD [2]. Here, we examined the expression of the two variants of *RET* mRNA in a case control study of stage-1 A^+^AD patients treated at Mayo Clinic between 1994 and 2007 (see Materials and Methods). Cases were classified as patients who died in less than 3 years after surgery (n= 28) and controls were patients who survived more than 5 years after surgery (n=38). A gap in years after surgery was included between cases and controls to minimize the possibility of overlap between aggressive tumors (cases) and non-aggressive (controls) tumors. Transcript variant 2 (RET51) had a significant negative association with the OS (p = 0.0057) with an AUC of 0.71 (Figure 1A). On the other hand, transcript variant 4 (RET9) was marginally predictive of OS (p = 0.046, Figure 1B) with an AUC of 0.68. These data suggest that between the two variants, the mRNA corresponding to the long RET has a better association with the OS.

### Silencing RET decreases the invasiveness of A^+^AD cells

**Figure 2:**
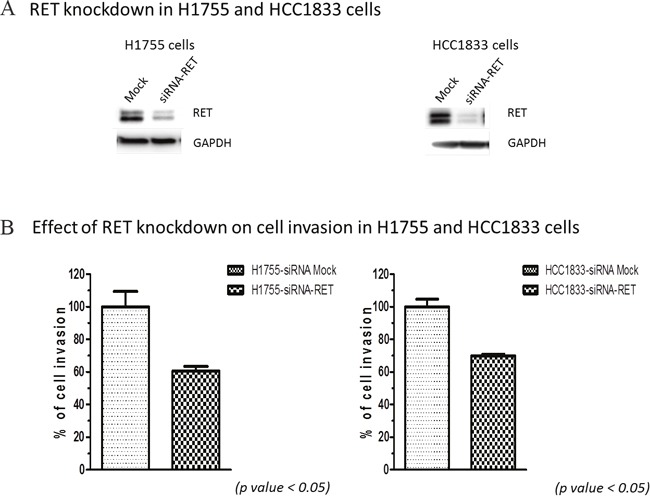
Effect of RET silencing on cell invasion in lung AD cells **(A)** RET silencing in H1755 and HCC1833 cells by siRNA. **(B)** Cell invasion of lung AD cells assessed by measuring their ability to invade through the polycarbonate membrane that mimics the extracellular matrix (ECM). Silencing RET using siRNA decreased cell invasion through polycarbonate membrane in both; HCC1833 and H1755 cells by almost 40%. These results were statistically significant as calculated by the unpaired *t-test* (p < 0.05).

To investigate the role of RET in cellular functions, we performed cell invasion and cell cycle analyses with HCC1833 and H1755 A^+^AD cells. Our results showed that knocking down RET reduced invasion by almost 40% in both cell lines (Figure 2). We also examined contributions of RET to apoptosis by measuring the number of cells in sub- G_0_/G_1_ phase of cell cycle using flow cytometry. Fewer than 3.5% of cells transfected with RET siRNA in both cell lines were undergoing apoptosis (Supplementary Figure 1). The apoptotic rates in un-transfected cells were even smaller. Also, no significant effects on cell proliferation were observed by RET siRNA knock down in both cell lines (data not shown). In contrast, ASCL1 expression had a positive association with cell proliferation (Supplementary Figure 2). Taken together, these results demonstrate that RET primarily influences the invasive properties of A^+^AD cells.

### EGFR inhibitors are selectively cytotoxic in A^+^AD cells

**Figure 3:**
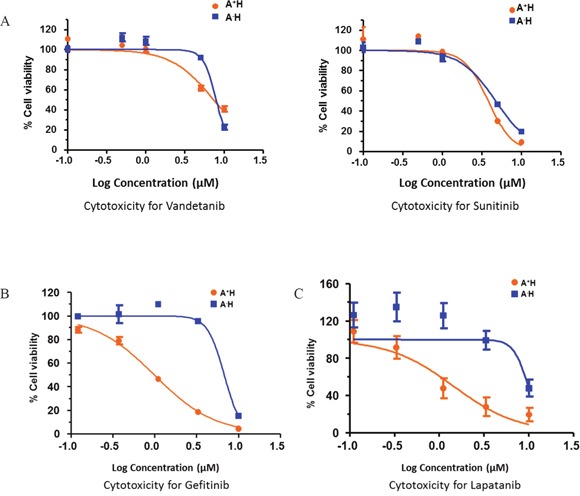
Drug response curves of lung AD cell lines for various receptor tyrosine kinase inhibitors (RTKIs) Knocking down ASCL1 made HCC1833 lung AD cells resistant to EGFR inhibitors. ASCL1 was silenced in HCC1833 as described in “Materials and Methods”. Plots are dose response curves to assess the effect of RET specific inhibitors vandetanib and sunitinib **(A)**, and EGFR inhibitors gefitinib **(B)** and lapatanib **(C)**.

To identify potential therapeutic options for A^+^AD patients, we examined the influence of various tyrosine kinase inhibitors that would selectively target A^+^AD cells compared to AD cells with low ASCL1 (A^−^AD). HCC1833 lung AD cells were stably transfected with either empty vector, which allowed cells to retain high endogenous levels of ASCL1/RET (A^+^H), or with ASCL1-shRNA which caused a robust reduction in endogenous levels of ASCL1 and RET (A^−^H) [2]. Cytotoxicity assays were performed on these cell lines using various tyrosine kinase inhibitors. In A^+^H cells, the known RET inhibitors vandetanib and sunitinib had no or at best a small selective cytotoxicity (Figure 3A). In contrast, we observed close to 10 fold enhanced cytotoxicity by the EGFR inhibitor gefitinib in A^+^H (Figure 3B). An additional five EGFR inhibitors were examined; lapatanib, pelitinib, dacomitinib, afatinib and canertinib. Among these lapatanib was the most selective with over 10 fold increased cytotoxicity in A^+^H compared with A^−^H cells (Figure 3C). Pelitinib and dacomitinib had moderate selectivity (supplementary Figure 3) and afatinib and canertinib were not selective (data not shown). Overall, these results suggested a possible interaction between EGFR and RET in A^+^AD cells.

### EGFR mediates phosphorylation of RET in A^+^AD cells

**Figure 4:**
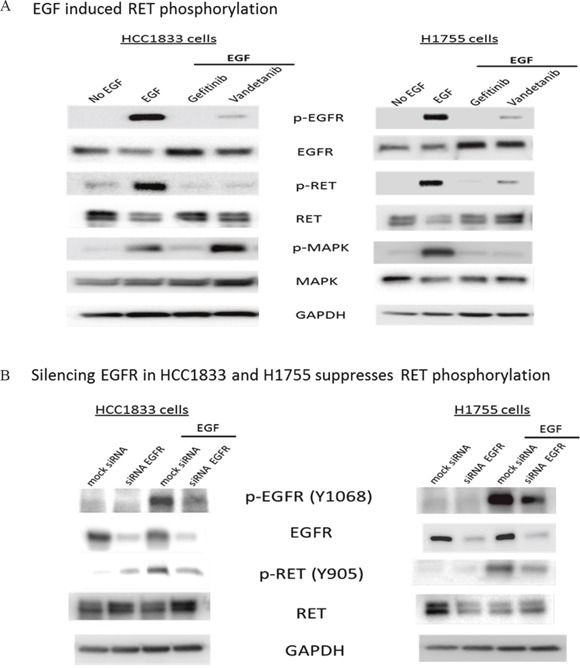
EGF induced phosphorylation of RET in HCC1833 and H1755 cells Both HCC1833 and H1755 cells were starved in serum free media for 24 hours followed by treatment with EGF (10ng/ml) for 15 minutes. For treatment with gefitinib and vandetanib, cells were starved overnight in serum free media followed by 24 hours treatment with 1μM gefitinib or vandetanib followed by stimulation with EGF as above. Proteins were collected and separated using 4-20% gradient polyacrylamide gel. The membrane was immunoblotted for EGFR, p-EGFR, RET and p-RET using respective antibodies as described in “Materials and Methods”. EGF induced phosphorylation of RET at Y-905 position in both HCC1833 cells and H1755 cells. This phosphorylation of RET at Y-905 position was significantly inhibited on treatment with gefitinib and vandetanib **(A)**. EGFR was silenced using siRNA in HCC1833 and H1755 cell lines and its effect on RET phosphorylation at Y-905 was assessed by Western blot. Silencing EGFR reduced RET phosphorylation upon EGF stimulation **(B)**.

To investigate the potential role of EGFR in the activation of RET, we examined HCC1833 and H1755 cells upon stimulation with EGF. Interestingly, we not only observed EGFR phosphorylation at Tyr1068, but also a strong band corresponding to RET phosphorylation at Tyr905. This EGF induced phosphorylation of RET and EGFR was inhibited by treatment with gefitinib in both cell line models (Figure 4A) and also upon silencing EGFR by siRNA (Figure 4B). The RET inhibitor vandetanib also reduced RET phosphorylation markedly, but to a lesser extent than gefitinib (Figure 4A). However, we observed increased MAPK phosphorylation in HCC1833 but not in H1755 on treatment with vandetanib followed by stimulation with EGF (Figure 4A). Taken together, these results provided evidence that EGFR mediates RET activation in A^+^AD cells.

### RET and EGFR interact only in the presence of EGF and predominantly through the long RET isoform

**Figure 5:**
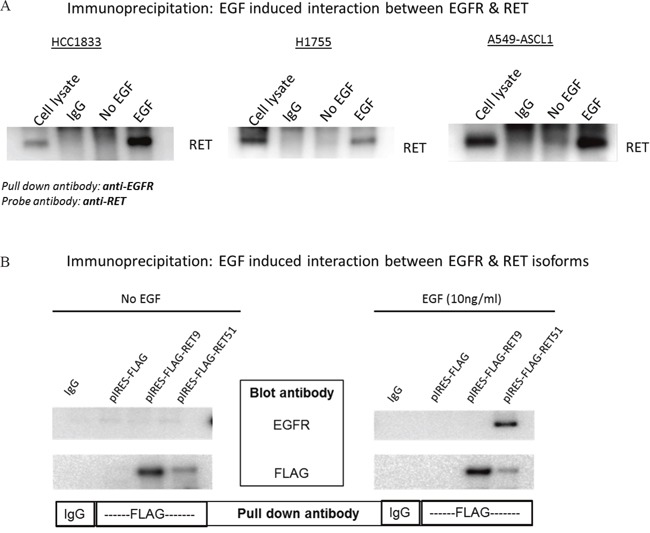
EGF induced interaction between EGFR and RET HCC1833 and H1755 cells were stimulated with EGF as mentioned in “materials and methods” section. Total protein lysate was incubated with EGFR antibody to precipitate EGFR. The immunoprecipitated protein was separated using 4-20% gradient polyacrylamide gel and the membrane was immunoblotted using anti-RET antibody. EGF stimulates interaction between RET and EGFR in HCC1833, H1755 and A549-ASCL1 cells **(A)**. cDNA of human RET51 and RET9 gene cloned into pIRES with FLAG tag plasmid was transfected into HEK293 cells. The cells were stimulated with EGF as mentioned in “Materials and Methods” section and total protein lysate was collected. Lysed protein was incubated with anti-FLAG antibody; then separated using 4-20% gradient polyacrylamide gel followed by immunoblotting the membrane using anti-EGFR antibody. Results show clear binding between RET51 and EGFR on stimulation with EGF **(B)**.

We investigated the possibility of interaction between EGFR and RET by co-immunoprecipitaion (co-IP) using HCC1833, H1755 and A549-ASCL1 cells. HCC1833 and H1755 have high endogenous expression of ASCL1 and RET whereas ASCL1 was stably overexpressed in A549 cells which induced overexpression of RET (Supplementary Figure 4). Anti-EGFR antibody was used for IP followed by probing with anti-RET antibody. As shown in Figure 5A, binding between RET and EGFR was observed after EGF stimulation. This binding was disrupted in HCC1833 and H1755 cell lines using the EGFR inhibitor; gefitinib, but not in A549-ASCL1 cells (Supplementary Figure 5). Vandetanib, primarily a RET inhibitor disrupted this interaction only in H1755 cells (Supplementary Figure 5). The reverse strategy of co-immunoprecipitating with the anti-RET antibody and blotting using the anti-EGFR antibody was not successful potentially due to the antibody binding site being blocked by the EGFR-RET interaction. Therefore, to confirm the above observation, as well to learn about potential preference of EGFR to interact with specific RET isoform, we induced expression of human RET51 and RET9 by transfecting the FLAG tagged cDNA constructs into HEK293 cells (see Methods). After IP with anti-FLAG antibody and probing using anti-EGFR antibody, we observed clear interaction between EGFR and RET that occurred only upon EGF stimulation and this interaction was predominantly between EGFR and the long RET isoform (RET51) (Figure 5B).

### High levels of EGFR and RET associate with a poor prognosis in A^+^AD microarray datasets

**Figure 6:**
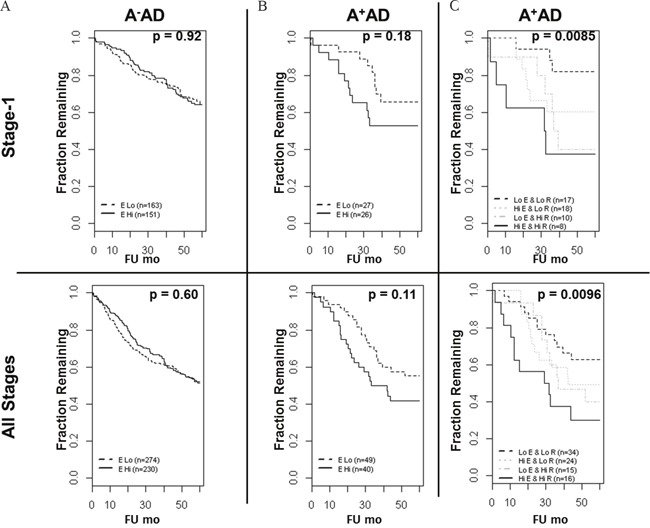
Overall survival of AD patients based on the ASCL1, RET, and EGFR status **(A)** In A^−^AD, EGFR mRNA was not predictive of overall survival. **(B)** In A^+^AD, there was a trend toward shorter OS in patients with high EGFR status which was statistically not significant. **(C)** Importantly, A^+^AD with high EGFR and RET had significantly shorter OS compared with A^+^AD with low EGFR and RET in both stage-1 (hazard ratio = 5.6) and in all stages (hazard ratio = 2.8) of tumors. Reported p-values are based on logrank tests.

Previously, we reported an inverse association between the OS and *RET* mRNA expression in a compendium of microarray data in stage-1 and all stages of A^+^AD [2]. Here, we examined the *EGFR* mRNA expression in A^−^AD and A^+^AD and its prognostic significance in our previously described microarray dataset (see Methods). There was approximately a two-fold down regulation of EGFR in A^+^AD compared with A^−^ AD which was highly significant (p < 10^−7^) (Supplementary Figure 6). Functional significance of EGFR down regulation in A^+^AD is unclear. We performed survival analyses including clinical parameters (see Methods and Supplementary Table 1). In A^−^AD, EGFR mRNA was not associated with the OS. In A^+^AD on the other hand, EGFR association with OS was significant in a cohort of all stage tumors (p = 0.027) and marginally significant in stage-1 tumors (p= 0.055). Interestingly, when we added RET to the models that included EGFR, the association with OS was considerably enhanced (p-value decreased from ≤ 0.055 to ≤ 0.0032, Supplementary Table 1). For illustration by the KM curves, we dichotomized samples based on the mean expression of EGFR and RET in A^−^ and A^+^AD. Clinical parameters in A^+^AD were insignificant and were excluded from dichotomized analyses (see Methods). We observed similar results as above (Figure 6). Notably, A^+^AD tumors with high EGFR and RET had significantly shorter survival times than A^+^AD tumors with low EGFR and RET (p < 0.01) (Figure 6C). These results provided further evidence that EGFR and RET contribute cooperatively to an aggressive phenotype in A^+^AD.

## DISCUSSION

Our results demonstrated for the first time an EGFR mediated activation of RET in A^+^AD cells, which was dependent upon EGF stimulation. This activation was diminished by treatment with both the EGFR inhibitor gefitinib and RET inhibitor vandetanib. On further investigation, we demonstrated an EGF induced interaction between EGFR and RET that was abrogated by both EGFR and RET inhibitors, but to differing degrees in the three cell lines, potentially stemming from differences in the origins of the cell line models and their individualized neoplastic properties from the mutation profiles in each tumor model. While gefitinib was effective in disrupting EGF induced EGFR/RET interaction in the HC1833 and H1755 cell lines, vandetanib disrupted the binding only in H1755. This suggests that the process by which EGFR-RET interaction occurs is intricate and may involve several other partners, which could be important to gauge patient drug response. Support for additional effectors are indicated in Figure 4A, with the differential effects on the MAPK pathway in HC1833 and H1755 cells (p-MAPK band) following EGF activation in the presence of the RET inhibitor vandetanib. The absence of either drug blocking this interaction in A549-ASCL1^+^ could stem from this cell lines origin through stable overexpression from an ASCL1 transcript and not through a natural mechanistic progression as in the HC1833 and H1755 cell lines. Independent support for the interaction between EGFR and RET was demonstrated upon co-IP of transfected FLAG tagged RET constructs. This experiment further identified the long RET (RET51) isoform in the interaction, demonstrating important regulatory motifs in the c-terminal of the protein.

Previously, we demonstrated that levels of *RET* mRNA in tumors from A^+^AD patients had significant association with the overall survival (OS) in a large cohort of stage-1 AD. As EGFR inhibitors had high selective cytotoxicity in A^+^AD cells compared with A^−^AD cells, we examined adding EGFR to this RET model for predicting OS in stage-1 and all stages of A^+^AD [2]. High EGFR, in addition to high RET, was found to be a strong indicator of poor prognosis in A^+^AD (Figure 6 and Supplementary Table 1). Taken together, these findings suggest that A^+^AD patients may benefit from treatment with EGFR inhibitors even in the absence of EGFR or RET mutations. Our findings may also have clinical implications in subtypes of breast cancers which also have elevated expression levels of the wild type RET protein [5–7].

Our case control data suggests that the long RET isoform (RET51) has a stronger association with the OS than the short isoform (RET9) (Figure 1). This is consistent with the findings of Richardson *et. al*. [8], suggesting a more efficient presentation on the plasma membrane resulting in prolonged signaling through the downstream MAPK pathway by RET51 compared to RET9. Along these lines, we have found intense membrane staining by RET IHC to be associated with shorter survival in AD tumors (supplementary Figure 7). Interestingly, we also found that RET51 binds EGFR significantly stronger than RET9 (Figure 5B). Interactions between EGFR and chimeric forms of RET found in 30-50% of sporadic medullary thyroid cancers (RET/PTCs) have been reported by Croyle *et. al*. [9]. However, Croyle found that these interactions were independent of the c-terminal moiety of RET which is inconsistent with our data and could be due to the different EGFR binding organization between RET fusion proteins in thyroid cancers and wild type RET in A^+^AD.

Recently, three independent groups reported recurrent genomic rearrangements involving the *RET* locus in 1-2% of lung AD, predominantly in young patients who were light or non-smokers [10–12]. These events are likely non-overlapping with the over-expression of wild type *RET* in 10-20% of A^+^AD which are mostly limited to smokers [2]. A recurrent rearrangement event was additionally reported involving the generation of *KIF5B-RET* chimeric proteins [10]. Similar to their counterparts in 30-50% of sporadic medullary thyroid cancers [13], these fusion proteins have constitutive tyrosine kinase activity. Several ongoing clinical trials are investigating the effectiveness of tyrosine kinase inhibitors, including vandetanib and sunitinib known for higher specificity to RET, in treating lung cancers with RET rearrangements. In our cell culture experiments, we did not identify strong preferential sensitivity to these drugs in A^+^AD compared with A^−^AD cells. Interestingly, the data from Croyle *et. al*. also suggest that thyroid cancer cells carrying RET fusion proteins are sensitive to EGFR inhibitors. These data could also suggest that A^−^AD patients with RET fusion positive tumors might benefit from treatment with EGFR inhibitors.

To summarize, our data suggest a cooperative interaction between EGFR and RET in lung AD with neuroendocrine differentiation characterized by the expression of ASCL1. Between the two isoforms, the long RET isoform was found to have a more prominent role in the EGFR/RET interaction and also the mRNA corresponding to the long RET51 isoform had a stronger association with the OS. Our data also identified EGF induced activation of RET mediated through EGFR in A+AD cells which was blocked by EGFR inhibitors. Furthermore, A^+^AD with high EGFR and RET had significantly shorter OS than tumors with low EGFR and RET. These findings can lead to alternative therapeutic strategies constituting EGFR inhibitors in patients with A^+^AD.

## MATERIALS AND METHODS

### Measurement of mRNA expression using Nanostring

We used a case control design in stage-1 A^+^AD patients treated at the Mayo Clinic from 1994 to 2007. Cases referred to patients who died in 3 years after surgery and controls were patients who survived at least 5 years after surgery. ASCL1 expression status (A^+^ or A^−^) were based on ASCL1 mRNA by Nanostring. To enrich A^+^ samples, FFPE blocks (n=432) were stained for ASCL1 [2] and cases and controls mostly from IHC positive samples were selected for Nanostring measurements. One hundred and fifty nano-gram of extracted RNA from each block in 5μl of RNase free water was added to 20 μl of master mix containing hybridization buffer and reporter probes. Just before putting the samples in a hybridization cycler pre-set at 65°C, 5μl of the capture probes was added to each reaction and mixed using a micropipette. Hybridization reaction was carried out overnight at 65°C and next day the samples were processed using nCounter Prep-station, as directed in the company manual. The code sets for the target genes used in the study are recorded in Table S2 in the supplementary material. All experiments were under an IRB approved protocol.

### Nanostring data processing

The R package “NanoStringNorm” was used to generate normalized data using a geometric mean of four reference genes ALAS1, CLTC, GUSB, and TBP. Selection of A^+^ AD was based on normalized expression above the first quartile and a count of at least 20 corresponding to ASCL1 in the raw data. A logistic regression was used to determine the association of the two RET variants with the case/control status and the ROC plot was generated using the “Epi” package in R.

### Cell culture

A549 (ATCC CCL-185) and NCI-H1755 (H1755 or ATCC CRL-5892) lung AD cell lines were purchased from American Type Culture Collection (ATCC) (Manassas, VA, USA). HCC1833, another lung adenocarcinoma cell line was purchased from Korean Cell Line Bank (Seoul, South Korea). A549 and HCC1833 cell lines were cultured in RPMI 1640 medium [Corning, (Waltham, MA, USA)] whereas H1755 cells were cultured in RPMI-1640 media [ATCC 30-2001 (Manassas, VA, USA)]. Both the media contained 10% fetal bovine serum (FBS) and Penicillin-Streptomycin (10,000 U/mL) [Gibco, (Carlsbad, CA, USA)]. HEK-293 cells were purchased from ATCC (ATCC CRL-1573) and cultured using Dulbecco's Modified Eagle Medium (DMEM) containing 10% FBS and Penicillin-Streptomycin (10,000 U/mL) [Gibco, (Carlsbad, CA, USA)]. All cells were grown in a humidified incubator set at 37°C containing 95% air and 5% carbon dioxide level.

### Stable transfection of ASCL1 cDNA or shRNA

A549-ASCL1 cells with stable ASCL1 over-expression were generated by a stable transfection of A549 cells with a lentiviral plasmid pTN1060 [14] from Addgene [Plasmid # 31781 (phASCL1-N106), Cambridge, MA, USA] carrying human ASCL1 cDNA. Single clones were selected and expanded in RPMI 1640 media containing 10% FBS, Penicillin-Streptomycin (10,000 U/mL) and 10μg/ml of Blasticidin. Expression of ASCL1 was confirmed by western blotting using anti-ASCL1 antibody [BD Biosciences, (San Jose, CA, USA; cat# 556604)] (Supplementary Figure 4)

Stable transfection of HCC1833 cells with ASCL1 shRNA is previously described [2]. Briefly, HCC1833 cells transfected either with empty vector or ASCL1 shRNA were cultured in RPMI 1640 media containing 10% FBS, Penicillin-Streptomycin (10,000 U/mL) and 1μg/ml of Puromycin [2]. All cells were grown in an incubator set at 37°C in humidified atmosphere containing 95% air and 5% carbon dioxide level.

### siRNA transfection and western blotting

HCC1833 and H1755 cells were transfected with either control or RET siRNA [Qiagen, (Valencia, CA, USA; cat#SI02224985)] using Lipofectamine RNAiMAX reagent [Invitrogen, (Carlsbad, CA, UA)]. Cells were cultured in 6 well plate and transfected with siRNA following manufacturer's protocol. Volume of reagents was adjusted so that the final concentration of RET siRNA was 50 pmol. Cells were lysed using NETN buffer containing phosphatase and protease inhibitors and total proteins were collected 72 hours after transfection.

For drug treatment all cell lines were starved overnight. Cells were then treated with either 1μM gefinitib or vandetanib for 24 hours followed by EGF stimulation for 15 minutes. Proteins were then extracted using NETN buffer as mentioned above.

cDNA of human RET 51 and RET 9 was cloned in the modified vector pIRES2-EGFP (expressing protein with FLAG tag) kindly provided by Dr. Zhenkun Lou [Mayo Clinic, (Rochester, MN, USA)] [15]. HEK-293 cells were cultured in a 100 mm dish and 5μg of either empty vector or RET 51(p-RET51) or RET 9 (p-RET9) plasmid was transfected using Lipofectamine 2000 [Invitrogen, (Carlsbad, CA, USA)] reagent. Protein samples were collected with or without EGF stimulation 72 hours after transfection as above.

For western blotting, 20μg of total protein lysate was separated using 4-20% gradient polyacrylamide gel [Biorad, (Hercules, CA, USA)] and the membrane was blotted using either anti-EGFR (catalog # 4267), anti-phosphorylated EGFR (Tyr1068) (catalog # 3777), anti-RET (catalog # 14556), anti- phosphorylated RET (Tyr905) (catalog # 3221), anti-MAPK (catalog # 4695) or anti-phosphorylated MAPK antibody (catalog # 4370) [Cell Signaling Technology, (Danvers, MA, USA)].

### Co-immunoprecipitation

Proteins were collected from H1755, HCC1833 and A549-ASCL1 cells after respective treatments mentioned above. Total proteins were then incubated with anti-EGFR antibody overnight at 4°C with shaking to precipitate the EGFR complex. Immunoprecipitated complex was separated using 4-20% gradient polyacrylamide gel and the membrane was blotted using anti-RET antibody.

To investigate the interaction between RET51 or RET9 with EGFR, proteins were collected from HEK-293 cells transfected either with p-RET51, p-RET9 or empty vector with or without EGF stimulation. Total proteins were then incubated either with mouse monoclonal anti-FLAG tag antibody [Sigma Aldrich, (St. Louis, MO, USA)] or mouse IgG (control) overnight at 4°C with shaking. Immunoprecipitated complex was separated using 4-20% gradient polyacrylamide gel and the membrane was blotted using anti-EGFR antibody.

### Cell invasion assay

Cell invasion assay was performed using Cytoselect™ 24-Well Cell Migration and Invasion Assay (8μm, Colorimetric Format) kit by Cell Biolabs Inc. [(Catalog #CBA-100-C), San Diego, CA, USA]. Cells were transfected with either mock or RET siRNA. Twenty four hours after transfection, cells were suspended in no serum media at the concentration of 150,000 cells per 300μl and added to the inserts. To study the effect of gefitinib or vandetanib on cell invasion, cells were starved for 24 hours and then added to the inserts along with 1μM gefitinib and vandetanib. The inserts were then placed in wells containing 500μl of media containing 10% FBS. Cells were incubated at 37°C for 48 hours. After 48 hours, media in the inserts was removed and cells were carefully stained using the cell staining solution. After washing off excess staining solution, stained cells were extracted using the extraction buffer and OD was measured at 560nm using GloMax plate reader [Promega, (Madison, WI, USA)]. All experiments were performed in triplicates.

### Cell cycle analysis

Cell cycle analysis by flow cytometry followed the protocols by Cecchini [16]. Briefly, 2×10^6^ per ml of cells for each sample were collected and washed with PBS and spun at 300g for 5 minutes to remove the residual culture medium and trypsin. Cells were fixed on ice by incubation in 1% paraformaldehyde solution for an hour, washed with PBS, spun at 300g for 5 minutes at 4°C, and suspended in 0.5 ml of ice-cold PBS. Then, 4.5ml of ice-cold 70% ethanol was added drop wise to the cells followed by storage at -20°C overnight. Next day, cells were centrifuged at 300g for 5 minutes to remove ethanol followed by washing with 2 ml of PBS. Cells were then suspended in 0.5ml of propidium iodide staining buffer containing RNase followed by incubation at 37°C for 30 minutes in dark. Finally, cells were centrifuged to remove the buffer, re-suspended in 1ml of PBS and analyzed using a flow cytometer.

### Cell proliferation assay

Cells were counted using Countess II Automated Cell Counter [Invitrogen, (Carlsbad, CA, USA)] and 4000 viable cells in 100μl solution were plated in 96 wells. Cell proliferation after 24, 48 and 72 hours were performed by measuring emitted luminescence using a GloMax plate reader [Promega, (Madison, WI, USA)] after addition of 20 μl of Cell-Titer Glo reagent [Promega, (Madison, WI, USA)] and incubation at room temperature for 10 minutes in the dark.

### Survival analyses in the microarray datasets

The microarray dataset used to examine the expression and the prognostic significance of EGFR and RET was described previously [2]. Briefly, we combined a Mayo lung AD dataset (n = 132) with 3 other microarray datasets that had follow up information. These included the Director's Challenge dataset [17] (n = 420), Kune dataset (GEO dataset GSE10245, n = 40), and Hou dataset (GEO dataset GSE19188, n = 45). The combined set included 593 samples of which 367 were stage-1 ADs. Threshold for ASCL1 status (+ or -) was chosen using 209988_s_at probeset at Log_2_ intensity of 8. RET and EGFR analyses used 215771_x_at and 201984_s_at probesets, respectively. Available clinical parameters were stage, gender, and smoking status. Tumor stage was categorized as either low (stages 1 and 2) or high (stages 3 and higher). In A^+^AD of all stages, gender was significant (p = 0.05). On the other hand, stage did not reach statistical significance most likely because the number of high stage tumors was limited (n=15). Also, all A^+^ADs were from smokers [2]; therefore smoking status was not included. In the analysis that included all stages of A^−^AD, significant parameters were stage (p < 10^−15^) and gender (p=0.02), but gender was insignificant when both gender and stage were included. Reported values for survival analyses (Supplementary Table 1B) were adjusted for stage (A^−^AD) and gender (A^+^AD). In all analyses of stage-1 tumors gender was not significant. To generate KM plots (Figure 6), EGFR thresholds in A^+^ and A^−^ AD were selected based on the mean EGFR expression in these tumors and samples were categorized into “High” or “Low” EGFR groups. Similarly, RET threshold in A^+^AD was based on the mean RET expression in these tumors. A^−^AD had mostly undetected expression of RET [2] and therefore RET was not included in the survival analyses. In A^−^AD (Figure 6A), EGFR status was not associated with the OS before and after adjusting for clinical parameters. Also, in comparing A^+^AD with high EGFR and RET against A^+^AD with low EGFR and RET, clinical parameters were not significant. Therefore, reported values in Figure 6 did not include clinical parameters. These analyses were by the “survival” package in R.

## SUPPLEMENTARY FIGURES AND TABLES


