# Supplementary material for: EGFR mediates activation of RET in lung adenocarcinoma with neuroendocrine differentiation characterized by ASCL1 expression
Source: Oncotarget. 2017 Feb 24;8(16):27155–65. doi: 10.18632/oncotarget.15676 (PMC5432325; doi:10.18632/oncotarget.15676)
Supplement: Supplementary file 1 [file oncotarget-08-27155-s001.pdf]

EGFR mediates activation of RET in lung adenocarcinoma with neuroendocrine differentiation characterized by ASCL1 expression

SUPPLEMENTARY FIGURES AND TABLES

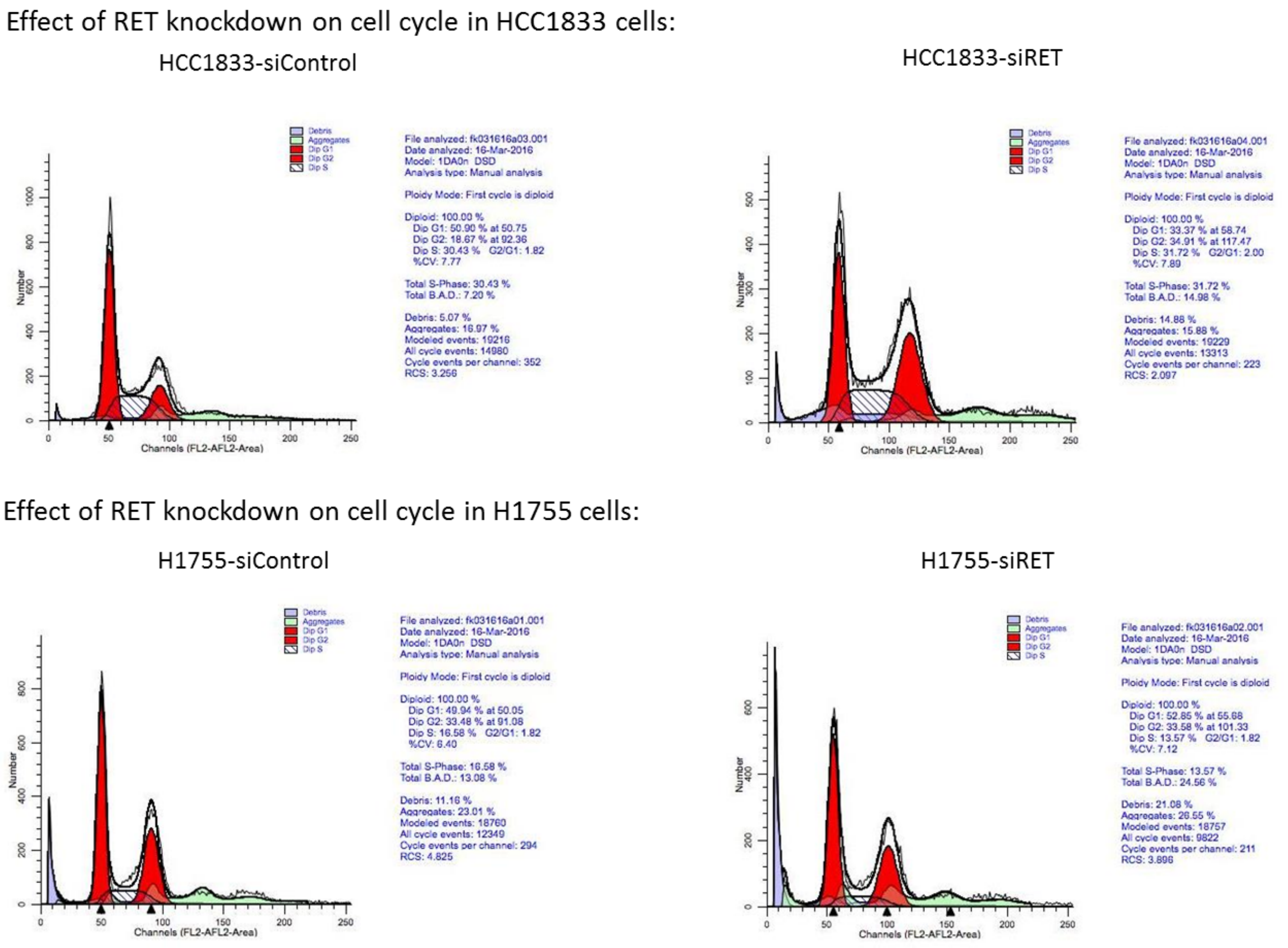

Supplementary Figure 1: Effect of RET manipulation on cell cycle analysis and cell proliferation. RET was transiently silenced in HCC1833 and H1755 cell lines using siRNA as described in “Materials and Methods”. Cell cycle analysis was performed using flow cytometry by counting the percentage of propidium iodide (PI) stained cells in each phase of cell cycle. Cell proliferation was assessed by measuring the luminescence (indicator of viable cells) emitted after addition of “Cell-titer Glo” reagent. Results indicate that in both HCC1833 and H1755 cells there was no significant effect on cell proliferation (data not shown).

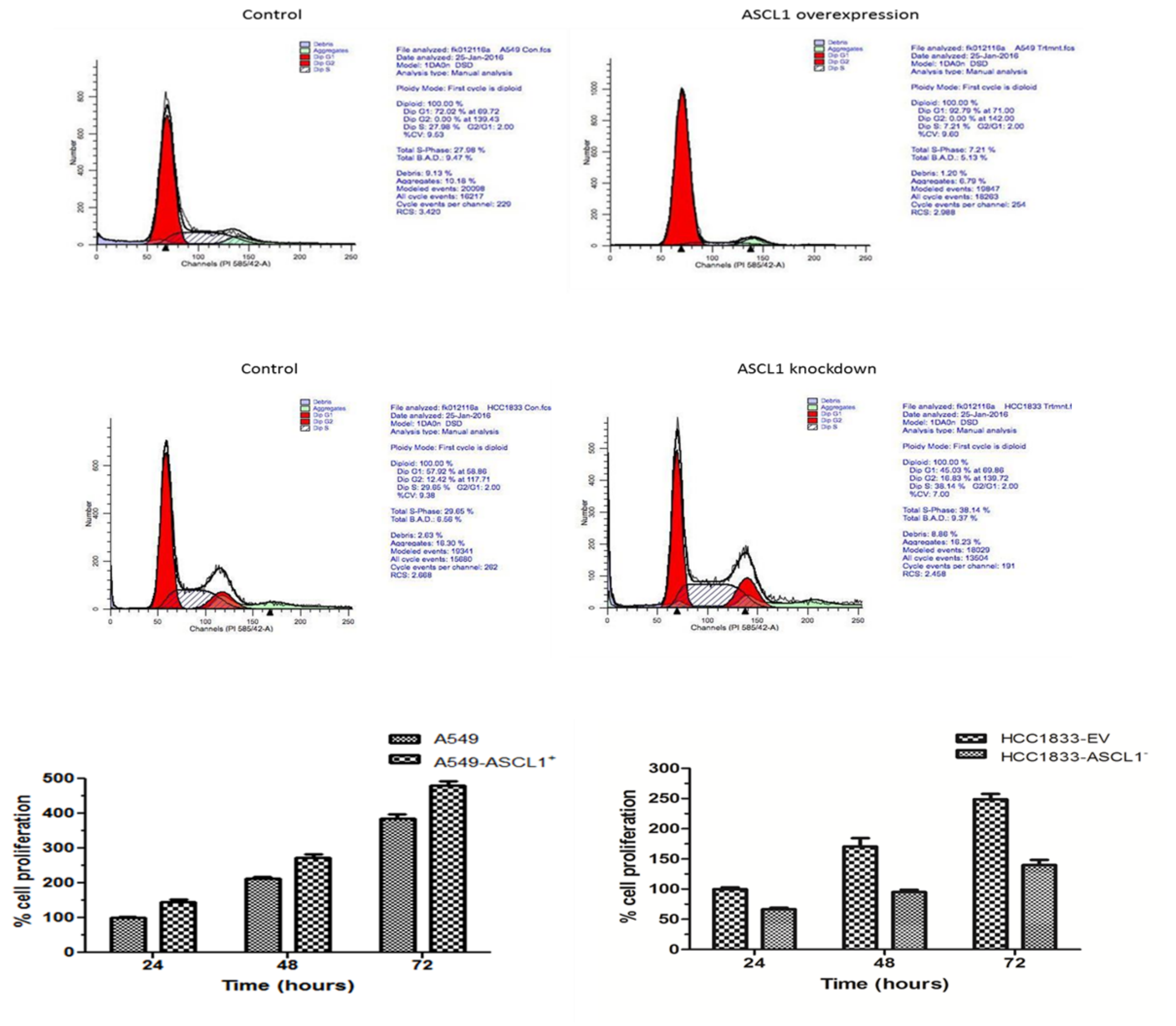

Supplementary Figure 2: Effect of ASCL1 manipulation on cell cycle analysis and cell proliferation. ASCL1 was stably overexpressed and silenced in A549 and HCC1833 cells, respectively. Cell cycle analysis was performed using flow cytometry by counting the percentage of PI stained cells in each phase of cell cycle. Cell proliferation was assessed by measuring the luminescence (indicator of viable cells) emitted after addition of "Cell-titer Glo" reagent. Overexpressing ASCL1 in A549 cells promoted cell proliferation. Furthermore, the number of cells in G_0/G_1 increased by almost 30% shortening the S phase (Figures 2A and 2C upper panel). Silencing ASCL1 in HCC1833 reduced cell proliferation and decreased the number of cells in G_0/G_1 phase by 23% while the number of cells in S and G2/M phase combined increased indicating prolonged cell proliferation (Figures 2B and 2C lower panel).

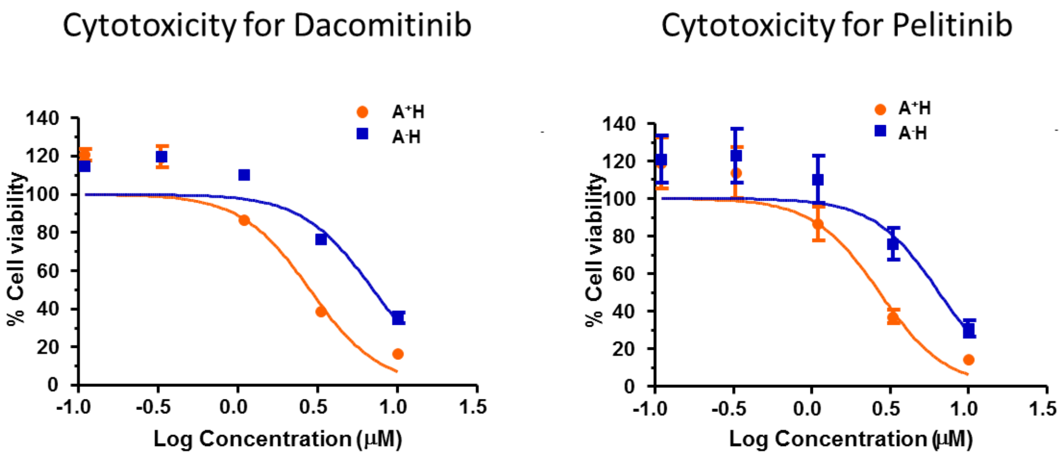

Supplementary Figure 3: Drug response curves of lung AD cell lines for various EGFR inhibitors; Knocking down ASCL1 made HCC1833 lung AD cells resistant to EGFR inhibitors. ASCL1 was stably silenced in HCC1833 as described in "Materials and Methods". Plots are dose response curves to assess the effect of EGFR inhibitors dacomitinib and pelitinib.

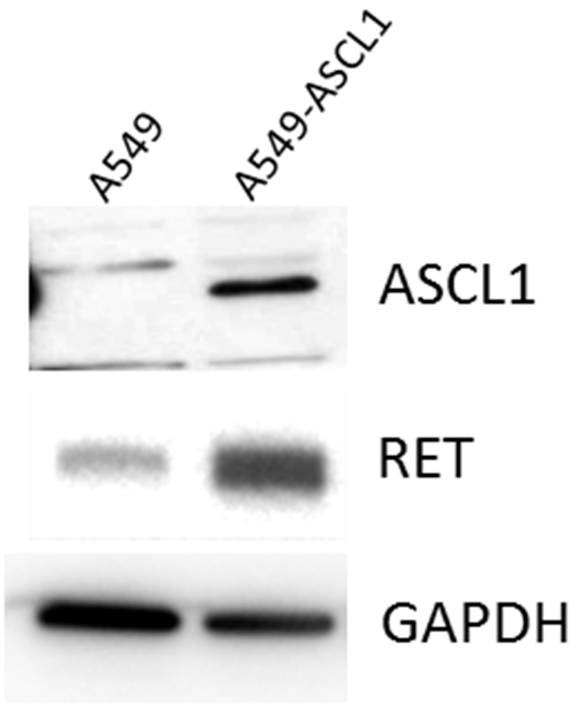

Supplementary Figure 4: ASCL1 overexpression in A549 cells: ASCL1 cDNA was stably transfected in A549 cells as described in "Materials and Methods". Total protein lysate was separated using 4-20% gradient polyacrylamide gel and the membrane was immunoblotted using either anti-ASCL1 or anti-RET antibody. Overexpression of both ASCL1 and RET in A549 cells was confirmed.

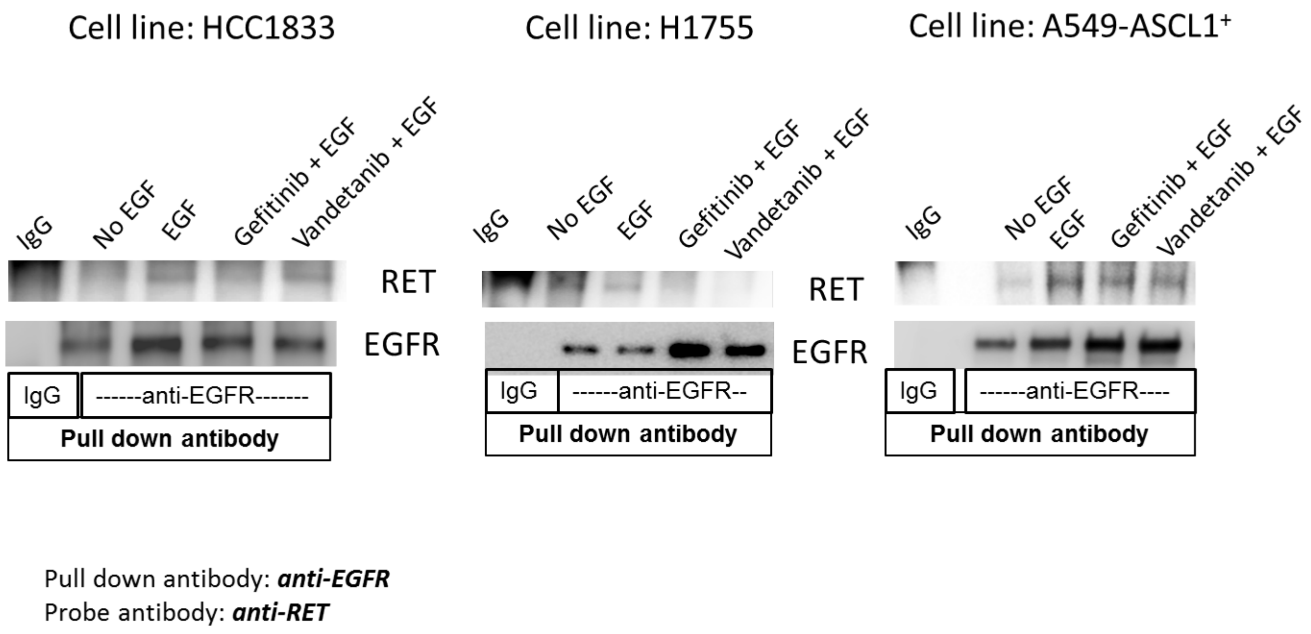

Supplementary Figure 5: Effect of gefitinib and vandetanib treatment on EGFR-RET interaction. HCC1833, H1755 and A549-ASCL1 cells were subjected to respective treatments as mentioned in “Materials and Methods” section. Total protein lysate was incubated with EGFR antibody to precipitate EGFR. The precipitated protein was separated using 4-20% gradient polyacrylamide gel and the membrane was immunoblotted using anti-RET antibody. Gefitinib disrupts interaction between RET and EGFR in HCC1833 and H1755 cells whereas vandetanib disrupts this interaction only in H1755 cells. Neither gefitinib nor vandetanib disrupts interaction between RET and EGFR in A549-ASCL1 cells.

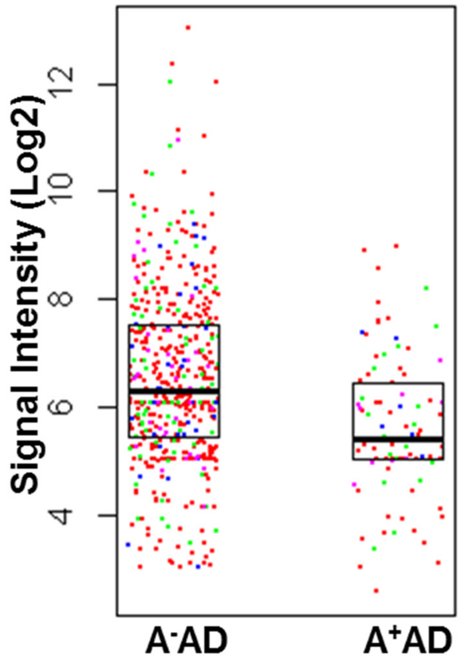

Supplementary Figure 6: EGFR expression in A⁺ and A⁻AD. Red, green, blue, and pink are data points in Director Challenge, Mayo, Kune, and Hou datasets, respectively in all stages of lung AD. There was approximately a two-fold down regulation of EGFR in in A⁺AD (n=89) compared with A⁻AD (n=504). The difference in EGFR expression between the two sets was highly significant ($p < 10^{-7}$).

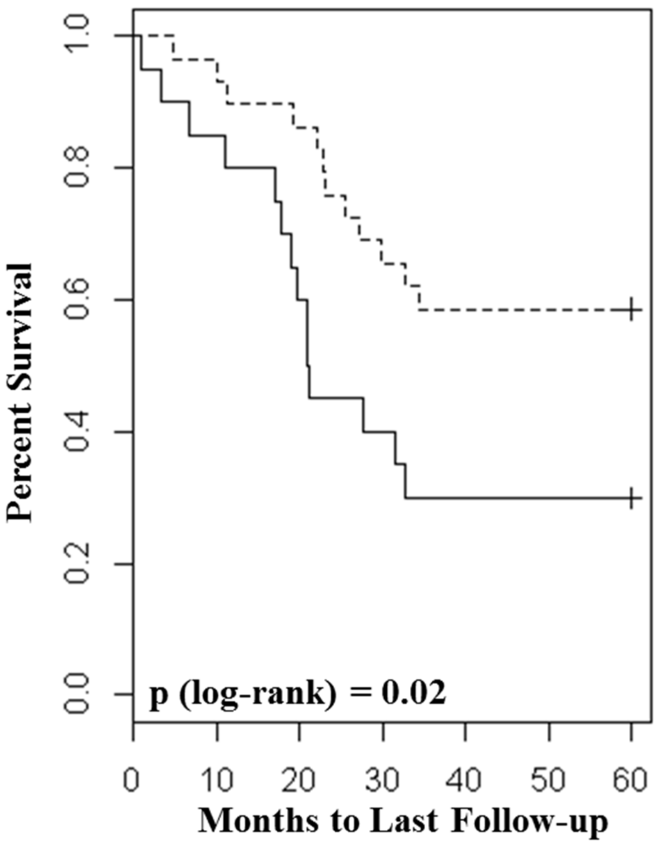

Supplementary Figure 7: Intense membrane staining by RET IHC is associated with poor overall survival. Stage-1 adenocarcinoma tumors (n=49) were stained by RET antibody (sigma HPA008356) and the 5-year overall survival (OS) in tumors with intense membrane staining (intensity 3+, solid line) was compared with tumors with no or weaker membrane staining (dashed line). Association with OS was estimated by a long-rank test using "coxph" function in survival package in R.

Supplementary Table 1: A high EGFR in addition to a high RET is a poor prognostic indicator. Analysis used A^+AD in a compendium of microarray datasets reported previously (Oncogene. 2014 Jul 17; 33 (29):3776-83)

Supplementary Table 1A: Influence of EGFR and RET mRNA expression level on the OS of Stage-1 AD

A^- AD			A^+ AD		
**p-value (log-rank)**	**EGFR p-value**	**coxph Model**	**RET p-value (HR)**	**EGFR p-value (HR)**	**p-value (log-rank)**
0.46	0.46	EGFR		0.055 (1.35)	0.053
NA		RET	0.0084 (1.40)		0.0059
NA		RET + EGFR	0.0056 (1.43)	0.036 (1.40)	0.0026

Reported p-values and hazard ratios (HR) are for stage-1 (Supplementary Table 1A).
Entries include p-values and hazard ratios (HR, in parenthesis).

Supplementary Table 1B: Influence of EGFR and RET mRNA expression on the OS of AD (including all stages)

A^- AD			A^+ AD		
**p-value* (log-rank)**	**EGFR p-value**	**coxph Model**	**RET p-value (HR)**	**EGFR p-value (HR)**	**p-value** (log-rank)**
$<10^{-15}$	0.60	EGFR		0.027 (1.27)	0.012
NA		RET	0.017 (1.25)		0.0097
NA		RET + EGFR	0.024 (1.23)	0.035 (1.25)	0.0032

All stages (Supplementary Table 1B) of lung AD.
Entries are p-values and hazard ratios (HR, in parenthesis).
* Adjusted for stage ($p < 10^{-15}$)
** Adjusted for gender ($p < 0.04$)

Supplementary Table 2: Sequence for probes used for identification of RET and ASCL1 mRNA using Nanostring™

GENE NAME	NUCLEOTIDES TARGETED	PROBE SEQUENCE
RET (NM_020975.4)	4571-4670	CAATGACAATGACCAAGGACTGCTACACCTCTGATTACAA<br>TTCTGATGTGAAAAAGATGGTGTTTGGCTC<br>TTATAGAGCCTGTGTGAAAGGCCCATGGAT
RET (NM_020630.4)	3991-4090	TTTCCCTTACCCACCTTCAGGACGGTTGTCACTTATGAAGTCAGTG<br>CTAAAGCTGGAGCAGTTGCTTTTTGAAAG<br>AACATGGTCTGTGGTGCTGTGGTCT
ASCL1 (NM_004316.3)	1651-1750	ATAGTAACTCCCATCACCTCTAACACGCACAGCTGAAAGTTCTTGCTCGG<br>GTCCCTTCACCTCCTCGCCCTTTCTTAAAGTGCAGTTCTTAGCCCTCTAG
ASCL1 (NM_004316.3)	1244-1343	GAGGAGCAGGAGCTTCTCGACTTCACCAACTGGTTCTGAGGGGCTCGGCC<br>TGGTCAGGCCCTGGTGCGAATGGACTTTGGAAGCAGGGTGATCGCACAAC
ASCL1 (NM_004316.3)	953-1052	CGCAACCGCGTCAAGTTGGTCAACCTGGGCTTTGCCACCCTTCGGGAGCA<br>CGTCCCCAACGGCGCGGCCAACAAGAAGATGAGTAAGGTGGAGACACTGC
TBP (NM_001172085.1)	588-687	ACAGTGAATCTTGGTTGTAAACTTGACCTAAAGACCATTGCACTTCGTGCCC<br>GAAACGCCGAATATAATCCCAAGCGGTTTGCTGCGGTAATCATGAGGA
GUSB (NM_000181.1)	1351-1450	CGGTCGTGATGTGGTCTGTGGCCAACGAGCCTGCGTCCCACCTAGAATC<br>TGCTGGCTACTACTTGAAGATGGTGATCGCTCACACCAAATCCTTGGACCC
ALAS1 (NM_000688.4)	1616-1715	GGGGATCGGGATGGAGTCATGCCAAAAATGGACATCATTTCTGGAACACTT<br>GGCAAAGCCTTTGGTTGTGTTGGAGGGTACATCGCCAGCACGAGTTCTC
CLTC (NM_004859.2)	291-390	GGGTATCAACCCAGCAAACATTGGCTTCAGTACCCTGACTATGGAGTCTGAC<br>AAATTCATCTGCATTAGAGAAAAAGTAGGAGAGCAGGCCCAGGTGGTA
